# Hypofractionation trials and radiobiology of prostate cancer

**DOI:** 10.18632/oncoscience.347

**Published:** 2017-04-14

**Authors:** Sarah Gulliford, Emma Hall, David Dearnaley

**Affiliations:** The Institute of Cancer Research, London, UK

**Keywords:** prostate, hypofractionation

Evidence has accumulated suggesting that prostate cancer (PCa) may be particularly sensitive to radiation fraction size [1]. This has considerable implications for the delivery of radical radiation treatments suggesting that shorter treatments using higher dose/fraction schedules might improve the therapeutic ratio and make treatment more convenient for patients as well as using radiotherapy resource more effectively. The fraction sensitivity of cancer and normal tissues is usually described by a linear quadratic equation. The *ratio* α/β of the linear (α) and quadratic (β) terms is a useful measure of the “curviness” of dose effect curves. Early responding tissues and many cancers typically have a high α/β ratio, making them sensitive to total dose but with a small fractionation effect. Late responding tissues and perhaps PCa have a low α/β ratio, demonstrating increased cell survival at low dose/fraction and significantly greater toxicity/effect at high dose/fraction. Four large randomised controlled trials testing modest hypofractionation for localised PCa have reported efficacy and side effect outcomes within the last year [2-5]. The largest trial, CHHiP, which included 3216 patients [2] compared standard fractionation (SFRT) using 2.0Gy daily fractions (f) (total dose 74Gy) with two experimental hypofractionated (HFRT) schedules using 3.0Gy/f (total doses of 60Gy and 57Gy). The trial used a non-inferiority design and demonstrated that HFRT at 60 Gy was non–inferior to SFRT. Five year disease control rates defined by biochemical (PSA)/clinical failure free outcome were for HFRT (60Gy) 90.6% (95% confidence intervals 88.5–92.3) compared with SFRT 88.3% (86.0–90.2) (hazard ratio 0.84, (95% CI: 0.65–1.07)); treatment related toxicities were low and similar. A complementary study design was used in the PROFIT trial [3] which included 1206 patients and compared SFRT using 2.0Gy/f (total dose 78Gy) with the same HFRT schedule of 3.0Gy/f (total dose 60Gy). HFRT was again shown to be non-inferior to SFRT with identical 21% biochemical/clinical failure rates at 5 years. In PROFIT gastro-intestinal side effects were increased in the SFRT group compared with HFRT group probably due to the higher SFRT dose given compared with CHHiP. Both investigator groups suggested that HFRT (60Gy/20f in 4 weeks) could be considered a new standard of care [6]. In contradistinction authors of the HYPRO study [4] came to different conclusions testing dose escalated HFRT. 804 patients received either SFRT 78Gy in 2Gy daily fractions or HFRT giving 64Gy in 3·4Gy fractions but importantly treating with three fractions per week and therefore protracting overall treatment time (OTT). The gain in tumour control was smaller than might have been expected from the radiobiological model (HFRT 80.5% vs. CFRT 77.1%) and not statistically significant. The relatively unfavourable side effect profiles may be due to the higher HFRT doses delivered. The trial failed to demonstrate either superior efficacy or non-inferior side effects for HFRT. These 3 studies were undertaken in predominantly intermediate and high risk localised PCa. 1092 men were included in RTOG trial 0415 [5] which tested the non-inferiority of HFRT in low risk PCa. Daily schedules of SFRT (73.8Gy/1.8Gyf) were compared with HFRT (70Gy/2.5Gyf). The cumulative incidence of biochemical recurrence at 5 years was 8% and 6% in SFRT and HFRT groups respectively which met the protocol-specified non-inferiority criterion but late gastrointestinal and genitourinary side effects were increased with HFRT. There was no certain improvement in the therapeutic ratio.

Together the 4 trials provide a rich source of data to further explore the radiobiology of prostate cancer response and hint that there may be a time factor resulting from tumour repopulation [1]. Independent estimation of the α/β value can be derived for each of the 4 trials by adjusting for the small differences in outcomes in the SFRT and HFRT trial groups. In this case the estimates of α/β are 1.3Gy (PROFIT), 1.7Gy (CHHiP 57Gy) and 1.9Gy (CHHiP 60Gy) and the considerably higher values of 3.5 Gy (HYPRO) and 6.9Gy (RTOG-0415). Repopulation can be modelled using OTT and Tk - the number of days from the start of treatment when repopulation is assumed to begin. A comprehensive approach to estimating these unknown variables is currently being developed [7]. However preliminary results assuming a proliferation rate (pr) of 0.31Gy [1] indicate that including a “time factor” improves the data fit. Estimates of the α/β ratio increase and cluster between 3.8-5.4 Gy except for RTOG-0415 which is an outlier at 31.5Gy. We speculate that this may be because of the low proliferation rate of low grade PCa. All these estimates are associated with wide confidence intervals and should be treated with considerable caution since although they are derived from large clinical trial cohorts, it is not possible to resolve 3 unknown variables (α/β, Tk, pr) which may vary between the PCa risk groups included in these data. Although the clinical trial results are adequately robust to recommend a change in prostate cancer fractionation to 60Gy in 20 fractions much remains to be learnt about PCa radiobiology which will have significant impact on the development of more extreme hypofractionation schedules [8].
